# *Hafnia alvei* Pneumonia: A Rare Cause of Infection in a Patient with COVID-19

**DOI:** 10.3390/microorganisms9112369

**Published:** 2021-11-17

**Authors:** Lucía Méndez, Jorge Ferreira, Cátia Caneiras

**Affiliations:** 1 Microbiology Research Laboratory on Environmental Health (EnviHealthMicro Lab), Institute of Environmental Health (ISAMB), Faculty of Medicine, Universidade de Lisboa, 1649-028 Lisboa, Portugal; lucia.mendez@chedv-min.saude.pt; 2 Pulmonology Department, Centro Hospitalar Entre o Douro e Vouga, 4520-221 Santa Maria da feira, Portugal; jorge.ferreira@chedv-min.saude.pt; 3 Institute of Preventive Medicine and Public Health, Faculty of Medicine, Universidade de Lisboa, 1649-028 Lisboa, Portugal; 4 Microbiology and Immunology Department, Faculty of Pharmacy, Universidade de Lisboa, 1649-003 Lisboa, Portugal

**Keywords:** COVID-19, ventilator-associated pneumonia, bacterial co-infection, *Hafnia alvei*, gram-negative, critical care, Portugal

## Abstract

Herein, we describe a case report of a critically ill patient, a 48-year-old man without comorbidities admitted to the hospital with a serious type 1 (hypoxemic) respiratory insufficiency and confirmed diagnosis of COVID-19. After 5 days with invasive mechanical ventilation, the patient developed a bacterial co-infection, namely a pneumonia by *Hafnia alvei*, requiring the last line of respiratory support: extracorporeal membrane oxygenation (ECMO). Subsequently, his clinical situation gradually stabilized, until he was discharged from the hospital on day 61, being accompanied in ambulatory consultation by the physical medicine and pulmonology department during the post-COVID-19 recovery. *H. alvei* is a Gram-negative bacterium that is rarely isolated from human specimens and is rarely considered to be pathogenic. However, COVID-19 disease can cause substantial organ dysfunction and can be associated with bacterial secondary infections which can favor the emergence of rare infectious diseases by uncommon microorganisms.

## 1. Introduction

Patients with SARS-CoV-2 infection can develop coronavirus disease 2019 (COVID-19) [1]. Most people with COVID-19 develop only mild or uncomplicated illness, while approximately 14% develop severe illness requiring hospitalization and oxygen, and 5% may require invasive mechanical ventilation. In severe cases, COVID-19 can be complicated by acute respiratory distress syndrome (ARDS), sepsis and septic shock, and multi-organ failure [2,3,4]. Several opportunistic infections were reported among COVID-19 patients [5], despite that its real impact is not completely understood. Some studies report *Aspergillus* spp., *Candida* spp., *Cryptococcus neoformans*, *Pneumocystis jiroveci* (*carinii*), *Cytomegalovirus*, *Herpes simplex virus*, *Mycobacterium tuberculosis*, and *Toxoplasma gondii* as the main opportunistic infections [5], while others studies report scarce (<10%) fungal, viral and bacterial co-infections [6]. The most common bacteria described were *Mycoplasma pneumonia*, *Pseudomonas aeruginosa*, and *Haemophilus influenzae* [6], despite the increasing reports of multidrug-resistant Gram-negative pathogens [7,8,9].

*Hafnia alvei* is a Gram-negative enteric and oropharyngeal bacillus and usually is nonpathogenic. It is rarely isolated from human specimens and is infrequently considered to be pathogenic [10,11,12]. However, this bacterium can be responsible for serious infections in neonates [13] and adults, especially in hospitalized patients with underlying chronic diseases, subjected to invasive procedures or even under antibiotic treatment [14]. Moreover, despite the limited studies worldwide, *H. alvei* was previously related to pneumonia clinical condition [10,12,15,16,17,18]. Herein we report a case of a critically ill patient with SARS-CoV-2 and *Hafnia alvei* respiratory co-infection.

## 2. Case Presentation

A 48-year-old Caucasian patient, a civil construction worker with no medical history, was admitted to the emergency room our hospital for fever, dyspnea, and hypoxemia. On the objective examination, he was vigilant but listless, with adequately answers to simple questions, without gross neurological deficits. The vital signs were as follows: blood pressure 80/50, cardiac frequency 100 ppm, respiratory frequency 40, and oxygen saturation 90% with oxygen at 15 L/min by high concentration mask. He had symmetrical auscultation, no snoring, no wheezing, and no crackling. At the physical evaluation, it was observed that the patient was of thin structure, without edema, but with peripheral cyanosis. The following auxiliary diagnostic tests were requested at admission (Table 1): (i) blood cultures and SARS-CoV-2 research; (ii) arterial blood gas analysis with oxygen at 15 L/min by high concentration mask which results were as follows: pH 7.46; partial pressure of carbon dioxide (PCO2) 30; partial pressure of oxygen (PO_2_) 61; sodium (Na) 133; potassium (K) 3.4; calcium (Ca) 1.22; chloride (Cl) 106; glucose 159; lactate 2; BE (base excess) −2.5 mmol/L; bicarbonate (HCO_3_[−]) 22 mEq/L; (iii) chest X-ray that suggested bilateral infiltrates; and (iv) blood analyzes for which the following results were obtained: without anemia, thrombocytopenia, and without renal and/or hepatic dysfunction; elevated C-reactive protein (CRP). Moreover, at admission day, blood cultures, urocultures, and tracheal aspirate were also requested. The detection of antigens for *Legionella* and *Influenza* viruses were requested too. A relative neutrophilia was observed (86%) but all analyses to detect microorganisms were negative.

Considering the chest imaging with bilateral infiltrates and the confirmation of the presence of the virus in oronasal swab by the microbiology laboratory, the patient had a diagnosis of pneumonia by SARS-CoV-2.

Due the severe patient clinical condition, endotracheal intubation and invasive mechanical ventilation were decided at the admission day, for which reason was send to the intensive care unit (ICU).

At the ICU, the patient was hemodynamically unstable with 5 points on the Acute Physiology and Chronic Health Evaluation (APACHE II), that is a severity score and mortality scale, and 27 points on the Social Physique Anxiety Scale (SPAS) II scale, a measurement instrument of physique anxiety.

From a neurological perspective, the patient was sedated for a Richmond Agitation Sedation Scale (RASS) scale of −5. The patient received intravenous fluid therapy. Renal function was normal, but due to hypernatremia and hyperchloremia identified at blood analysis, the patient undergoes potassium and phosphorus replacement treatment. At the hematological level, he was on pharmacological thromboprophylaxis with enoxaparin. The patient performed enteral feeding at a rate of 50 mL/h with good tolerance. However, due to a constipation gastrointestinal disorder the patient started treatment with bisacodyl, a stimulant laxative that works by increasing the amount of fluid/salts in the intestines

At admission day, the respiratory system of the patient was the most affected, so in addition to the immediate implementation of invasive mechanical ventilation in a controlled volume mode, the patient underwent periods of prone position and started systemic corticosteroid therapy with dexamethasone. Moreover, the patient started empirical antibiotic therapy with amoxicillin/clavulanic acid and azithromycin.

On the fourth day, feeding content was detected in the aspiration of the orotracheal tube, probably caused by the intestinal transit problems previously identified.

Between the day 1 and day 5, the patient began to have clinical significant improvement with a respiratory prognosis to evolve from “severe” (PaO_2_/FiO_2_ ratio <100) to “moderate” (PaO_2_/FiO_2_ ratio 200–100) [19], namely with a PaO_2_/FiO_2_ ratio going from 61 (at admission) to 133 (day 5) supported by biomarkers stabilization.

However, at day 5, the patient starts febrile, with an elevated c-reactive protein and high lymphocyte blood levels, indicative that the patient was dealing with an infection or other inflammatory condition. On the same day, due co-infection suspicious, blood, urine and aspirated bronchial secretions samples were collected and send to the laboratory.

Actually, aspirated bronchial secretions collected at fifth day, revealed moderate growth of Gram-negative bacilli leading to the identification of *Hafnia alvei* bacterium. No other infectious agents were identified in all samples tested. The antibiotic susceptibility testing of *H. alvei* was performed according to the 2020 European Committee on Antimicrobial Susceptibility Testing (EUCAST) clinical breakpoints - bacteria (v 10.0) and was susceptible to cefepime, ertapenem and gentamicin. Moreover, was susceptible, increased exposure to cefotaxime. *H. alvei* is intrinsically resistant to both ampicillin and amoxicillin/clavulanic acid.

Unfortunately, due to constraints associated with weekends and Christmas holidays (the hospital microbiology laboratory is not “24 hours a day, 7 days a week, 365 days a year”), the results were only known 5 days later (end of day 10).

Thus, due the high and persistent fever of the patient, the therapy was empirically changed at day 5 from amoxicillin/clavulanic acid and azithromycin (started at admission) to piperacillin/tazobactam beta-lactam/beta-lactamase inhibitor combination (that was maintained from day 5 to day 10 and changed at day 10 due to patient clinical worsening).

In fact, the ratio of the partial pressure of oxygen and inspired oxygen fraction of the patient was 133 on day 5 and, worryingly, this ratio decreased abruptly to 57 on day 10. On this day (day 10), the antibiotic therapy was again empirically altered to meropenem and linezolid. Finally, at the end of the day, the laboratory results were known and the antibiotic therapy was maintained, considering the expected susceptibility to carbapenem therapy. Due to the significative worsening of the ventilatory situation during this day, the patient was transferred to a tertiary-care hospital to perform an extracorporeal life support technique, namely extracorporeal membrane oxygenation (ECMO). He was on ECMO for 9 days (day 10 to day 19), and thereafter continued mechanically ventilated for additional 10 days (until day 29) through a tracheostomy cannula. After these days, the cannula was removed for closure by second intention and the patient has remained at spontaneous ventilation.

In the overall time, the patient was hospitalized for 61 days, 50 of which at the ICU. After discharge from the hospital, the patient remained with follow-up by the physical medicine and rehabilitation service to carry out the hospital protocol for post-COVID-19 respiratory rehabilitation. No new or recurrent infections were identified at home.

## 3. Discussion

To the best of the authors’ knowledge, this is the second worldwide case of co-infection by SARS-CoV-2 and bacterial co-infection by *Hafnia alvei*, un uncommon pathogen, and the first reported in Portugal.

The treatment of COVID-19 patients constitutes a very complicated challenge, especially among patients with severe disease. Immunosuppressive therapy has shown promising results for control of the cytokine storm syndrome in severe cases of COVID-19. However, it is well documented that immunosuppressive agents (e.g., corticosteroids) increase the risk of opportunistic infections [5]. In our case description, despite that it was a man without comorbidities, the patient with COVID-19 is admitted to the hospital with a serious hypoxemic respiratory insufficiency, starts corticosteroid therapy with dexamethasone, is transferred to the ICU, and initiates invasive mechanical ventilation treatment on the admission day that we believe has significantly contributed to increasing the risk of health care-associated infection (HAI) development.

The *H. alvei* was identified 5 days after hospital admission and at a time when the patient was having a good clinical progression, which leads us to believe that it was a HAI or ventilator-associated pneumonia [20] by an uncommon pathogen. The Center for Disease Control definition of health care-associated infection in the acute care setting deemed that an HAI was present if cultures were positive and obtained after 3 days of hospital admission [21]. Days to positive cultures was defined as time from day of admission to the day the culture was collected. There is still scarce published studies about predictors and outcomes of HAI in COVID-19 patients [22]. Another justification can be that, although the patient reported had a good tolerance to food, the fact that there was no intestinal transit during the 4 days following admission could have provoked vomiting, initially unnoticed and in large quantities because the balloon of the orotracheal tube did not function sufficiently as a barrier and aspirated food content, and the microorganism is subsequently detected in the usual bronchial lavage. Still, these facts demonstrates that a usually non-pathogenic microorganism can be a cause of pneumonia and thus of bacterial superinfection and severe progression of the disease.

At the time of admission, only inflammatory biomarkers could indicate superinfection, since the blood cultures and urine cultures requested were negative, thus supporting the justification for empirical antibiotic therapy. Given the worsening of the clinical situation and even after the detection of the isolation of *H. alvei*, our patient changed antibiotic treatment on two more occasions in order to broaden the spectrum of action. All three antimicrobial choices (amoxicillin/clavulanic acid and azithromycin; piperacillin/tazobactam and meropenem and linezolid) were empirically selected considering that the laboratory results are not obtained at the speed required to establish the most appropriate treatment. A rethinking of hours of operation of clinical microbiology laboratory is required to shorten time to accurate result reporting and to optimize patient care [23].

A previous study performed by the authors at the same hospital showed an increase in isolates of Gram-negative microorganisms in the last 5 years, an important factor when selecting empirical antibiotic therapy [24]. However, of relevance, the *H. alvei* is intrinsically resistant to amoxicillin/clavulanic acid and the piperacillin/tazobactam combination was not tested. It is important, whenever possible, to administer specific antibiotics to fight infections more effectively and to avoid promoting antimicrobial resistance. In this particular case, the microorganism was susceptible to the final antibiotic therapy administered, but strains of *H. alvei* with resistance to piperacillin-tazobactam due the production of *AmpC* enzymes and to last therapeutic lines, as carbapenems [25] (one of the last-line antibiotics to combat multi-resistant gram-negative bacteria) have already been described highlighting the potential relevance of this pathogen.

Initially, many cases of COVID-19 were treated as a bacterial co-infection associated with COVID without associated inflammatory markers. It is now known that a low proportion of patients with COVID-19 have bacterial co-infection (about 7% of hospitalized patients and 14% in ICU); fewer than in the previous influenza pandemics. The current evidence does not support the routine use of antibiotics in the management of confirmed COVID-19 infection [6]. Moreover, emphasize that the challenge of antibiotic-resistant bacteria has not get away with COVID-19 [26] and that the Gram-negative [7,8,9], namely the carbapenem-resistant pathogens, should remain a public-health priority [27,28,29].

Interestingly, the clinical case reported in Italy of *H. alvei* pneumonia under a COVID-19 patient described a severe disease progression, also with ECMO respiratory need, similar to our case. The authors conclude that COVID-19 influences the lung microbiota dynamics and favors the emergence of rare infectious diseases [15].

As learning points, we can emphasize: (1) COVID-19 places the patient in an immunocompromised situation that causes opportunistic agents to develop nosocomial infections; (2) Respiratory infections by food aspirations in the airway are a high risk of nosocomial infection especially in patients without airway defense mechanisms; (3) *H. alvei*, although rare, can cause nosocomial respiratory infections; and (4) it is important that whenever possible to administer targeted antibiotics in order to fight infections more effectively and avoid promoting antimicrobial resistance. Moreover, our case highlights the unique clinical challenges in managing patients with SARS-CoV-2 and bacterial co-infection by *H. alvei*, which seems to be associated with critical respiratory cases and deemed difficult-to-treat.

## 4. Conclusions

In conclusion, COVID-19 disease can favor the emergence of rare infectious diseases by uncommon microorganisms as *Hafnia alvei* leading to a major clinical challenge. An active surveillance of bacterial secondary infections should be promoted in order to assess the best treatment efficacy.

## Figures and Tables

**Table 1:** Clinical parameters at patient admission.

Auxiliary Diagnostic Tests	Results at Admission
SARS-CoV-2 research	Positive
Blood cultures, urocultures and tracheal aspirate	Negative
*Legionella* and *Influenza* viruses antigens	Negative
PaO_2_/FiO_2_	61
pH	7.46
Partial pressure of carbon dioxide (PCO_2_)	30
Partial pressure of oxygen (PO_2_)	61
Sodium (Na)	133
Potassium (K)	3.4
Calcium (Ca)	1.22
Chloride (Cl)	106
Glucose	159
Lactate	2
Base excess (BE)	−2.5
Bicarbonate (HCO_3_[−])	22
C-reactive protein (CRP)	Elevated
Chest X-ray	Bilateral infiltrates

## Data Availability

The data presented in this study are available on request from the corresponding author.
